# A Joint Pharmacometric Model of Iohexol and Creatinine Administered through a Meat Meal to Assess GFR and Renal OCT2/MATE Activity

**DOI:** 10.1002/cpt.3612

**Published:** 2025-02-25

**Authors:** Zhendong Chen, Qian Dong, Charalambos Dokos, Jana Boland, Uwe Fuhr, Max Taubert

**Affiliations:** ^1^ Department I of Pharmacology, Center for Pharmacology, Faculty of Medicine and University Hospital Cologne University of Cologne Cologne Germany

## Abstract

Accurately assessing glomerular filtration rate (GFR) from plasma creatinine concentrations is challenging in patients with unstable renal function. This study aimed to refine the understanding of creatinine kinetics for more reliable assessments of GFR and net creatinine tubular secretion (nCTS) via OCT2/MATE in humans. In a clinical study of 14 healthy volunteers, iohexol was administered intravenously as a reference GFR marker, and creatinine was introduced through a meat meal. A joint pharmacometric model was developed using dense plasma and urine sampling. Simulations were used to evaluate the effect of different creatinine volume of distribution (V_d_) values on GFR estimation after acute kidney injury (AKI) and to assess the impact of limited sampling strategies on GFR and nCTS estimation. Pharmacokinetic parameters for iohexol and creatinine aligned with reported values, but a lower V_d_ of 41% of total body weight and a nCTS fraction of 31% relative to overall creatinine clearance were observed. Commonly used equations based on single‐point creatinine measurement all overestimated GFR, with the Modification of Diet in Renal Disease (MDRD) equation performing best, followed by Chronic Kidney Disease Epidemiology Collaboration (CKD‐EPI) 2009 equation. Simulations demonstrate the effect of V_d_ estimate accuracy on detecting AKI from creatinine plasma concentrations only. Following low‐dose iohexol administration, a single plasma sample at 5 hours and a urine sample from 0 to 5 hours provided accurate estimates of both GFR and nCTS using the joint model and enabled adequate correction for incomplete urine collection. This approach shows promise for assessing renal transporter activity based on estimated nCTS.

**Study Highlights**

**WHAT IS THE CURRENT KNOWLEDGE ON THE TOPIC?**

Creatinine volume of distribution (V_d_) plays a key role in estimating unstable glomerular filtration rate and is typically assumed to be 60% of total body weight (TBW), but was estimated at 73.8% of TBW in a previous study. Apart from glomerular filtration, creatinine undergoes tubular secretion via OCT2/MATE and tubular reabsorption. The net creatinine tubular secretion (nCTS) accounts for 10–40% of its renal excretion and has the potential for assessing renal transporter activity.

**WHAT QUESTION DID THIS STUDY ADDRESS?**

What is the true value of V_d_, and to what extent does it affect the estimation of unstable renal function? Additionally, how many samples are required to accurately estimate both GFR and nCTS following a low‐dose iohexol administration?

**WHAT DOES THIS STUDY ADD TO OUR KNOWLEDGE?**

A joint pharmacometric model of iohexol and creatinine was developed and validated, confirming key pharmacokinetic parameters but identifying a lower V_d_ of 41.3% of total body weight and a higher nCTS fraction of 31% relative to creatinine clearance. The model demonstrated that varying V_d_ values can introduce significant bias in GFR estimation. Using a single plasma sample at 5 hours and a urine sample from 0 to 5 hours after low‐dose iohexol administration, the joint model accurately predicted both GFR and nCTS.

**HOW MIGHT THIS CHANGE CLINICAL PHARMACOLOGY OR TRANSLATIONAL SCIENCE?**

The joint model of iohexol and creatinine can serve as an effective tool for estimating unstable GFR and nCTS, with nCTS potentially acting as a marker for assessing renal OCT2/MATE activity.

Estimated creatinine clearance (CrCL) and/or estimated glomerular filtration rate (eGFR) based on serum creatinine are commonly used in clinical laboratories to assess kidney function and guide drug dosing adjustments in chronic renal impairment.^1^ However, these estimates assume steady‐state conditions for creatinine formation and elimination, making them less reliable for patients with fluctuating renal function.^2^, ^3^, ^4^ Although measured CrCL using a collection of urine can be used when a steady state has not been reached and there is no change in kinetics, it assumes that the observed data are free of error and ignores common mistakes in urine collection.^5^ A study by van Acker et al. showed a circadian rhythm in GFR, with higher rates during the day and lower rates at night, which cannot be detected from relatively stable creatinine plasma concentrations.^6^ A slight post‐meal decrease in creatinine at 1.5 hours aligns with previously reported intra‐individual creatinine variation throughout the day.^7^, ^8^, ^9^ Therefore, GFR estimates based on a single time‐point creatinine plasma concentration under non‐steady‐state conditions are expected to be biased.

In contrast, compartmental nonlinear mixed‐effects modeling offers a feasible method to more accurately assess unstable CrCL and account for measurement errors. Ullah et al. proposed a compartmental creatinine model using plasma and urine creatinine data from critically ill patients, effectively describing renal function changes and outperforming standard methods.^10^ However, further evaluation of this model through an independent assessment of GFR is desirable before application. Additionally, the study assumed a creatinine volume of distribution (V_d_) of 60% of total body weight (TBW),^10^ which neglected variability and may result in biased estimates of other kinetic parameters. The magnitude of creatinine V_d_ is important because it determines how fast changes in CrCL are reflected by changes in plasma concentrations. Our prior analysis of published creatinine data in healthy volunteers, both with and without the ingestion of cooked meat,^11^ yielded an estimation of creatinine V_d_ close to total body water.^12^ It also provided an approximate estimate of the creatinine generation rate (CGR) consistent with reported values for healthy individuals. However, the absence of demographic information in this study limited further investigation into creatinine pharmacokinetics (PK).

Beyond the use of creatinine to assess global renal function, it may also serve as an endogenous marker to assess transporter activity. It is well‐known that creatinine is eliminated not only through glomerular filtration but also undergoes tubular secretion and reabsorption. The net contribution of tubular secretion, accounting for reabsorption, represents 10–40% of its total renal excretion.^13^ The transporter chain mediating this secretion has been identified as basolateral organic cation transporter 2 (OCT2) and apical multidrug and toxin extrusion proteins (MATE1 and MATE2‐K) by *in vitro* experiments as well as by clinical studies with the selective inhibitors cimetidine (MATEs), trimethoprim (MATEs), dolutegravir (OCT2) and pyrimethamine (OCT2 and MATEs).^14^ The effects of these inhibitors were much larger on renal metformin excretion, a drug also secreted by OCT2/MATE, because the fraction of metformin eliminated by tubular secretion is also much larger.^14^ Therefore, using overall renal excretion of creatinine to assess OCT2/MATE activity *in vivo* is not very informative.^15^ Still, when assessing net creatinine tubular secretion (nCTS) separately instead of overall excretion,^16^ creatinine may provide valuable information on renal OCT2/MATE activity including its inhibition by drug–drug interactions (DDIs).

The present study aims at improving the description of creatinine kinetics as a prerequisite for a more reliable creatinine‐based assessment of renal function and/or OCT/MATE activity in humans. To this end, we evaluated a dynamic creatinine model in a clinical trial in healthy volunteers by analyzing creatinine data from dense plasma and urine sampling and comparing the results to those obtained with iohexol as an independent GFR probe. The importance of correct creatinine V_d_ estimates was investigated by simulations. Finally, a limited sampling strategy was evaluated for simultaneous assessment of GFR and nCTS.

## METHODS

### Ethical approval

The clinical study was registered with the German Clinical Trials Register under the identification code DRKS00029908 and approved by the Ethics Committee of the Faculty of Medicine at the University of Cologne, Germany, on November 21, 2022 (No. 22‐1347_1). The study was conducted in accordance with Good Clinical Practice guidelines and the Declaration of Helsinki (64th WMA General Assembly, Brazil, October 2013).^17^ All volunteers provided informed written consent.

### Study design

#### Pilot study

A pilot study was conducted with two healthy adult participants, aged over 18 years with a body mass index (BMI) ranging from 18.5 to 30 kg/m^2^, to assess the feasibility of administering creatinine via cooked beef. Participants were enrolled after a pre‐screening evaluation of their health status. Key exclusion criteria included hypersensitivity to iohexol, previous reactions to contrast media, any relevant clinical or laboratory abnormality, concurrent medication use, smoking, drug addiction, and pregnancy or breastfeeding.

A two‐period crossover design was used, with participants receiving either 259 mg iohexol intravenously without food (test period) or 250 g cooked beef as a breakfast 25 minutes after intravenous administration of 259 mg iohexol (meat period). Beef was vacuum‐sealed and cooked at 70°C for 1.5 hours. Approximately 200 mg of cooked beef was collected in three aliquots for the measurement of creatinine content. Both participants received lunch (13.2 g non‐meat protein) and dinner (11.3 g non‐meat protein), and approximately 240 ml of plain water before and after urine collection. For pharmacokinetic analysis, blood samples were collected using EDTA‐K tubes at baseline and 0.17, 0.33, 0.5, 0.75, 1, 1.5, 2, 3, 4, 5, 6, 8, 10, 12, 16, 20, and 24 hours after iohexol administration in the non‐meat period. In the meat period, additional blood samples were obtained at 1.25, 2.5, 28, 32, and 36 hours post‐iohexol administration. Urine samples were collected during intervals of 0–2, 2–4, 4–6, 6–8, 8–10, 10–12, 12–16, 16–20, 20–24 hours in the non‐meat period, with additional samples at 24–28, 28–32, and 32–36 hours in the meat period. The wash‐out period was at least 7 days.

#### Main study

Twelve additional volunteers were included based on the same inclusion/exclusion criteria as the pilot study. The main study used a crossover design with six random sequences, where participants received three treatments: 3,235 mg iohexol under fasting conditions (reference period); 259 mg iohexol under fasting conditions (test period); and 3,235 mg iohexol with 250 g cooked beef (meat period). The inclusion of two iohexol dose levels aimed to assess a possible dose effect on iohexol clearance (IoCL); however, it is not the primary objective of this study and will be reported separately.^18^ The beef steak was minced in a blender and then vacuum‐sealed in a plastic bag. The minced beef was cooked in a water bath at 90°C for 1.5 hours to generate a significant amount of creatinine. All other conditions and procedures were identical with those employed in the pilot study. Quantification methods for iohexol and creatinine are available in the **supplementary materials**.

### Population pharmacokinetic analysis

Population pharmacokinetic (popPK) modeling was conducted using a nonlinear mixed‐effects approach with NONMEM version 7.4.0 (ICON Development Solutions, USA), Perl‐speaks‐NONMEM (PsN) version 5.3.0 (Uppsala University, Sweden), using the first‐order conditional estimation with interaction (FOCE‐I) method throughout model development. Post‐processing and plotting of NONMEM data were done using R version 4.3.0 (https://www.R‐project.org/). To compare nested models differing by one parameter, a statistical criterion of 3.84 in the objective function value (OFV) was used (equivalent to *P* < 0.05). The final model was evaluated using goodness‐of‐fit (GOF) plots, non‐parametric bootstrap analysis, and prediction‐corrected visual predictive check (pcVPC). Model estimates were compared to non‐compartmental analysis results (method described in **supplementary material**) to ensure no bias in parameter estimates.

The following assumptions were made for all participants: (1) no changes in the typical value of IoCL, CrCL, and CGR throughout the study; (2) iohexol and creatinine are solely eliminated via kidney; and (3) the circadian rhythms of iohexol and CrCL follow a consistent sine function pattern within a day.^6^

#### Joint population pharmacokinetic model for iohexol and creatinine

Based on prior knowledge, iohexol was modeled using a three‐compartment linear elimination model, and creatinine with a one‐compartment model.^10^, ^12^, ^19^ A first‐order process with lag time and a zero‐order process described creatinine uptake from meat and endogenous production, respectively.^12^ Different settings for creatinine dose assessment/bioavailability (F1) and creatinine V_d_ were evaluated for model performance and physiological plausibility (**Table** **1**). After establishing stable models for iohexol and creatinine, a joint model was developed where IoCL represented GFR and CrCL represented the sum of GFR and nCTS. The inter‐individual variability (IIV) and inter‐occasion variability (IOV) for PK parameters were modeled exponentially as the following equation: $\theta_{i}=\theta \times e^{\eta_{i}}$, where $\theta_{i}$ represents the value of the individual parameter value, θ represents the population point estimate, and $\eta_{i}$ is a normally distributed random variable with a mean of 0 and variance of $\omega^{2}$. Four independent proportional residual error models were used for plasma and urine data of iohexol and creatinine.

**Table 1:** Different settings for creatinine dose, bioavailability (F1), and volume of distribution (V_d_) as well as the parameter estimates and objective function value (OFV) from the respective models

Model	Dose input in the dataset	Mean (mg)	F1	Value	V_d_	Mean (L)	CL	CGR	OFV
	Setting		Setting		Setting		Value (mL/min)	Value (mg/h)	
1	Creatinine content in ingested beef	401	Fixed	100%	Estimated	76.6	136	67.9	3,809
2	Individual differences in creatinine excretion over 24 h between meat and non‐meat periods	335	Fixed	100%	Estimated	53.4	136	67.7	3,536
3	Individual differences in creatinine excretion over 16 h between meat and non‐meat periods	273	Fixed	100%	Estimated	44.7	135	67.8	3,502
4	Creatinine content in ingested beef	401	Estimated	61.9%	Fixed at “individual estimated total body weight × 0.6”	47.1	134	67.3	3,461
5	Creatinine content in ingested beef	401	Estimated	57.9%	Fixed at “individual estimated total body water”^22^	42.3	133	67.1	3,418
6	Creatinine content in ingested beef	401	Estimated	48.7%	Estimated	27.1	132	66.7	3,364

#### Covariate model

The circadian rhythm of both GFR and nCTS was first using the joint model based on the following equation:

$$\mathrm{CL}={\mathrm{CL}}_{\mathrm{TV}}\times \left(1+\sin\left(\frac{\text{time}}{\text{interval}\;}\times \pi \right)\times \theta_{\text{daytime}/\text{night}}\right)$$

where CL represents GFR or nCTS, ${\mathrm{CL}}_{\mathrm{TV}}$ is the typical value of GFR or nCTS, “time” was adjusted to start as 0, and “interval” is 14 hours for daytime and 10 hours for night. The food effect was assessed using a proportional model at 2‐hour intervals after a non‐meat protein meal. PK parameters were allometrically scaled to a TBW of 70 kg, using a power of 0.75 for GFR, nCTS, and inter‐compartmental clearances of iohexol (Q_p1_ and Q_p2_) and of 1.0 for iohexol central compartment volume (V_c_), creatinine V_d_, and iohexol peripheral compartment volumes (V_p1_ and V_p2_).^20^ In comparison, using other body size‐related covariates or estimating scaling factors for clearance and volume were subsequently assessed. The estimation of CGR was replaced by the Cockcroft‐Gault equation.^3^ Subsequent covariate analysis employed forward addition and backward elimination methods, with significance levels of 0.05 (ΔOFV <= −3.84) and 0.01 (ΔOFV <= −6.63), respectively. The effects of sex, age, height, TBW, BMI, lean body mass,^21^ estimated total body water,^22^ and plasma albumin concentration from laboratory test results on appropriate PK parameters were investigated. Covariate relationships were modeled based on the following equations:

(1) Continuous covariate:
$$P_{i}=P_{\mathrm{TV}}\times {\left(\frac{C_{\mathrm{ij}}}{\text{mean}\left(C_{j}\right)}\right)}^{\theta }$$
(2) Categorical covariate:
$$P_{i}=P_{\mathrm{TV}}\times \theta^{C_{\mathrm{ij}}}$$

where $P_{\mathrm{TV}}$ represents the typical value of parameter, $C_{\mathrm{ij}}$ represents the covariate value of participant i for parameter P, and $\text{mean}\left(C_{j}\right)$ represents the mean of covariate j in the investigated population.

### Simulation of creatinine profiles after presumed AKI

Creatinine plasma concentration profiles were simulated in 1000 virtual patients using the final model, with reductions in both GFR and nCTS evenly distributed from 25% to 75% across the population. Circadian rhythms of GFR and nCTS were not taken into account. The simulation data were subsequently re‐analyzed with different settings for creatinine V_d_ (from 41.3% to 73.8% of TBW).^10^, ^11^ For early diagnosis of acute kidney injury (AKI), estimating the changed GFR using first 1, 2, or 3 concentrations (assuming hourly sampling post‐AKI), as well as concentrations measured after 24 and 48 hours, were compared using the different V_d_ values described above. While using 41.3% as a reference, the relative error (RE) was calculated by the following equation:

$$\mathrm{RE}\left(\%\right)=\left(\frac{\text{Estimated changed}\;\mathrm{GFR}}{\text{True changed}\;\mathrm{GFR}}-1\right)\times 100\%$$

Creatinine plasma concentrations following a 75% reduction in both GFR and nCTS were simulated for an individual with median dataset covariates, using three different settings for creatinine V_d_. Based on the RIFLE (Risk, Injury, Failure, Loss of kidney function, and End‐stage kidney disease) classification for AKI definition, the times when plasma concentrations reach 1.5‐fold (risk), 2.0‐fold (injury), and 3.0‐fold (failure) of baseline level after AKI were calculate for each model.^23^

### Limited sampling strategy

Iohexol and creatinine plasma concentrations, along with amounts excreted in urine (A_e_), were simulated in 1,000 virtual individuals following a 259 mg intravenous dose of iohexol, using final model estimates of GFR and nCTS with a predefined 30% coefficient of variation. A random 10%–50% urine loss was applied to both iohexol and urine data over the 0–5 hours interval post‐dose, generating datasets (urine.loss) with incomplete urine collection. Various limited sampling strategies for estimating GFR were evaluated, based on 1 to 4 plasma samples collected at 10 minutes, 30 minutes, 2 hours, and 5 hours, with later samples included as sample numbers were reduced.^24^ The respective individual GFR estimates were then used to predict iohexol A_e_ over the 0–5 hours post‐dose interval. To account for incomplete urine collection, creatinine A_e_ was corrected by multiplying it by the ratio of predicted to incomplete iohexol A_e_, generating datasets (urine.corrected) with correction. Finally, iohexol plasma data, creatinine plasma data, and corrected urine data were used to estimate GFR and nCTS using the final model. The RE and root mean squared error (RMSE) of GFR and nCTS were calculated for each model.

## RESULTS

### Demographics

Fourteen participants, mean age 33 years (range: 23–48), were enrolled, including two in the pilot and 12 in the main study. The dataset includes 771 iohexol and 826 creatinine plasma concentrations, and 439 measurements for both iohexol and creatinine in urine. Observations with missing data (< 5%) were discarded. Detailed demographics and baseline characteristics are provided in **Table** **2**.

**Table 2:** Demographics and baseline characteristics of enrolled participants

Demographics/Characteristic	Male	Female	All	Range
	Mean (SD)	Mean (SD)	Mean (SD)	
Number	9	5	14	–
Age (years)	31 (6)	37 (8)	33 (8)	23–48
Height (cm)	183 (6)	169 (6)	178 (9)	163–196
Total body weight (kg)	86.2 (7.1)	64.5 (4.7)	78.5 (12.2)	59.1–95.8
Body mass index (kg/m^2^)	25.9 (2.1)	22.6 (0.9)	24.7 (2.4)	21.2–28.9
Body surface area (m^2^)	2.08 (0.10)	1.74 (0.09)	1.96 (0.19)	1.63–2.22
Plasma albumin (g/L)	45.7 (1.6)	45.4 (3.6)	45.6 (2.5)	40.0–50.0
Plasma creatinine concentration (mg/dL)^a^	1.00 (0.08)	0.73 (0.09)	0.90 (0.15)	0.60–1.17
Estimated creatinine generation rate (mg/h)^3^	78.4 (7.2)	48.0 (4.9)	67.3 (16.3)	41.0–92.6
Estimated lean body mass (kg)^21^	64.6 (3.7)	47.8 (3.7)	58.6 (8.9)	43.7–69.7
Estimated total body water (L)^22^	48.1 (2.6)	31.9 (1.7)	42.3 (8.1)	29.9–52.5
Estimated glomerular filtration rate (mL/min/1.73 m^2^)^27^	101 (9)	104 (12)	102 (10)	80.0–116

^a^ Creatinine plasma concentration was measured from a laboratory test at the screening visit.

### Population pharmacokinetic analysis

A three‐compartment model for iohexol and a one‐compartment model for creatinine fitted the data well. No further refinement was explored for the iohexol model since it provided a fully adequate description of the data. **Table** **1** lists the parameter estimates and OFV for models tested with different dose inputs (written in the datasets), F1, and creatinine V_d_. The creatinine amount in beef (mean: 401 mg) was higher than the differences in creatinine excretion between non‐meat and meat periods over 24 h (mean: 335 mg) or 16 h (mean: 273 mg), indicating less than 100% bioavailability. Meanwhile, a high correlation of 0.96 was observed between estimates of the calculated dose (dose input × F1) and creatinine V_d_. The creatinine model estimating both F1 and V_d_ yielded the best performance compared with other models, which all overestimated plasma concentrations during the meat period (**Figure** **S1**). This model provided estimates of F1 and V_d_ with low relative standard errors (RSE) of 9% and 6%, respectively, while the point estimates of CrCL and CGR remained stable across all tested models. IOV for CrCL and CGR was estimated at 3.1% and 3.3%, respectively, and was not included in the final model due to a lack of clinical significance.

In the joint model, all estimates of iohexol and creatinine PK parameters were consistent with those from separate models (**Table** **S1**). The schematic diagram of the joint model is presented in **Figure** **S2**. Circadian rhythms of GFR and nCTS were merged, resulting in better estimations with lower RSEs and enhanced model stability compared to estimating them separately. Subsequently, circadian rhythms during daytime and nighttime were assessed using the joint model, reducing OFV by 53.5 and 29.7, respectively. IIV on GFR, iohexol V_c_, nCTS, and creatinine V_d_ were estimated at 14.6%, 18.7%, 43.6%, and 18.4%, respectively. After including TBW as a covariate for all parameters by standard allometric scaling,^20^ IIV decreased to 11.8%, 14.5%, 32.3%, and 15.4%, respectively. Additional tests using other body size‐related covariates or estimating scaling factors for clearance and volume did not show significant improvement and thus standard allometric scaling was kept (**Table** **S2**). Replacing CGR with the Cockcroft‐Gault equation decreased the OFV by 21.5 and reduced the IIV for CGR by 17.6%, from 30.4% to 12.8%. Further covariate analysis found that sex significantly affected nCTS, reducing the OFV by 7.48.

GOF plots (**Figures** **S3** **and** **S4**) suggest that the final model fits the iohexol and creatinine data well overall, despite some slight overestimation in both creatinine plasma and urine data at early time points. Creatinine plasma data were better captured by the model with circadian rhythm. The pcVPC (**Figure** **S5**) also indicates a good fit for both iohexol and creatinine data. Estimates of all PK parameters in the final model, along with the 95% confidence intervals from bootstrap results, are listed in **Table** **3**. Fixed and random effects showed sufficient precision, with RSEs below 21.0% and 63.9%, respectively.

**Table 3:** Parameter estimates and bootstrap (*n* = 1,000) results for creatinine and iohexol

Parameters	Estimate	RSE (%)	95% confidence interval	CV (%)	Shrinkage (%)
Fixed effect					
Iohexol					
GFR (mL/min)	87.0	3.4	(81.0, 92.7)	–	–
V_c_ (L)	8.69	4.7	(7.91, 9.54)	–	–
Q_p1_ (L/h)	0.131	9.2	(0.107, 0.163)	–	–
V_p1_ (L)	1.15	5.2	(1.05, 1.33)	–	–
Q_p2_ (L/h)	4.01	8.0	(3.37, 4.78)	–	–
V_p2_ (L)	4.22	3.1	(3.93, 4.53)	–	–
Creatinine					
K_a_ (1/h)	1.71	13.3	(1.35, 2.19)	–	–
nCTS (mL/min)	39.7	10.2	(31.2, 46.8)	–	–
V_d_ (L)	28.9	6.5	(25.6, 33.4)	–	–
F1 (%)	52.3	5.2	(47.7, 58.4)	–	–
Lag time (h)	0.291	3.0	(0.277, 0.308)	–	–
CGR (mg/h)	(140–age) × TBW/72 × 0.85 (if female) × 60/100			–	–
Covariates					
Circadian rhythm during daytime (%)	3.70	21.0	(2.12, 5.72)	–	–
Circadian rhythm during nighttime (%)	8.42	17.7	(5.10, 11.1)	–	–
SEX on CTS^a^	0.627	15.0	(0.455, 0.857)	–	–
TBW on GFR, Q_p1_, Q_p2_, and nCTS	0.75 FIX	–		–	–
TBW on iohexol V_c_ and creatinine V_d_	1 FIX	–		–	–
Random effect (IIV)					
Iohexol					
GFR	0.0226	43.6	(0.00379, 0.0246)	11.9	0.1
V_c_	0.0211	38.2	(0.00585, 0.0390)	14.6	18.8
V_p1_	0.0121	63.9	(0.00164, 0.0259)	11.0	6.2
V_p2_	0.0113	33.8	(0.00304, 0.0187)	10.7	9.7
Creatinine					
nCTS	0.0506	56.7	(0.00333, 0.102)	23.1	10.6
V_d_	0.0211	40.7	(0.00284, 0.0375)	15.1	2.3
CGR	0.0163	31.2	(0.00710, 0.0278)	12.7	0.7
F1	0.00810	53.5	(0.00145, 0.0205)	10.4	22.8
Random effect (RV)					
Iohexol					
Plasma concentration	0.0171	18.4	(0.0118, 0.0240)	13.3	2.6
Excreted amount in urine	0.0617	27.2	(0.0330, 0.0970)	25.2	0.7
Creatinine					
Plasma concentration	0.00255	7.3	(0.00221, 0.00291)	5.1	1.8
Excreted amount in urine	0.0413	41.4	(0.0142, 0.0770)	20.5	1.2

GFR, iohexol clearance was assumed as GFR; nCTS, net tubular secretion part of creatinine clearance; V_c_, iohexol central compartment volume; Q_p1_, inter–compartment clearance between central and first peripheral compartment; V_p1_ first peripheral compartment volume; Q_p2_, inter–compartment clearance between central and second peripheral compartment; K_a_, apparent absorption rate; V_d_, creatinine volume of distribution; CGR, creatinine generation rate; F1, bioavailability; TBW, total body weight; RSE, relative standard error; CV, coefficient variance; IIV, inter‐individual variability; RV, residual variability.

^a^ SEX is a categorical covariate of 0 for male and 1 for female.

Final estimates for GFR (IoCL) and CrCL (GFR plus nCTS), scaled to the mean weight of 78.5 kg, were 94.8 mL/min and 138 mL/min, respectively. These estimates are consistent with non‐compartmental analysis results (results shown in **supplementary material**). In healthy participants, GFR accounted for approximately 69% of total CrCL, with 31% mediated by nCTS. Due to circadian rhythm, GFR and nCTS fluctuated between 104% and 91.6% of the mean over 24 hours. Creatinine V_d_ was estimated at 28.9 L, accounting for 41.3% of TBW in the investigated population.

**Figure 1:**
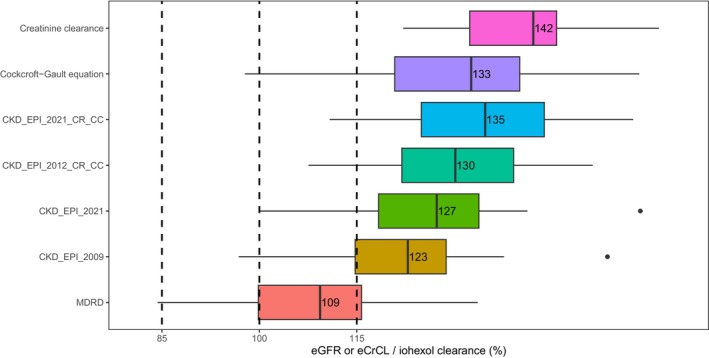
Comparison between post hoc estimates of iohexol clearance (“true GFR”) and creatinine clearance in the present model, estimated GFR (eGFR) or estimated creatinine clearance (eCrCL) calculated using commonly used equations (*n* = 14). All individual values were normalized by dividing eGFR/eCrCL by iohexol clearance. From top to bottom, the items are creatinine clearance using the popPK approach, eCrCL by Cockcroft‐Gault equation, eGFR by CKD‐EPI 2021 (based on creatinine and cystatin C), CKD‐EPI 2012 (based on creatinine and cystatin C), CKD‐EPI 2021 (based on creatinine only), CKD‐EPI 2009 (based on creatinine only), and MDRD equation.

Comparison of post hoc estimates of IoCL and CrCL with eGFR using different equations, including Cockcroft‐Gault equation,^3^ CKD‐EPI 2021 (creatinine and cystatin C),^25^ CKD‐EPI 2012 (creatinine and cystatin C),^26^ CKD‐EPI 2021 (creatinine only), CKD‐EPI 2009 (creatinine only),^27^ and four‐variable modification of diet in renal disease (MDRD) equation,^28^ is presented in **Figure** **1**. The eGFRs based on single time‐point concentration prior to administration from commonly used equations all overestimate GFR, with the MDRD equation performing best, followed by CKD‐EPI 2009. Conversely, CKD‐EPI 2021 (creatinine and cystatin C) and Cockcroft‐Gault equations provided the closest CrCL estimates.

### AKI simulation for different creatinine V_d_ values

**Figure 2:**
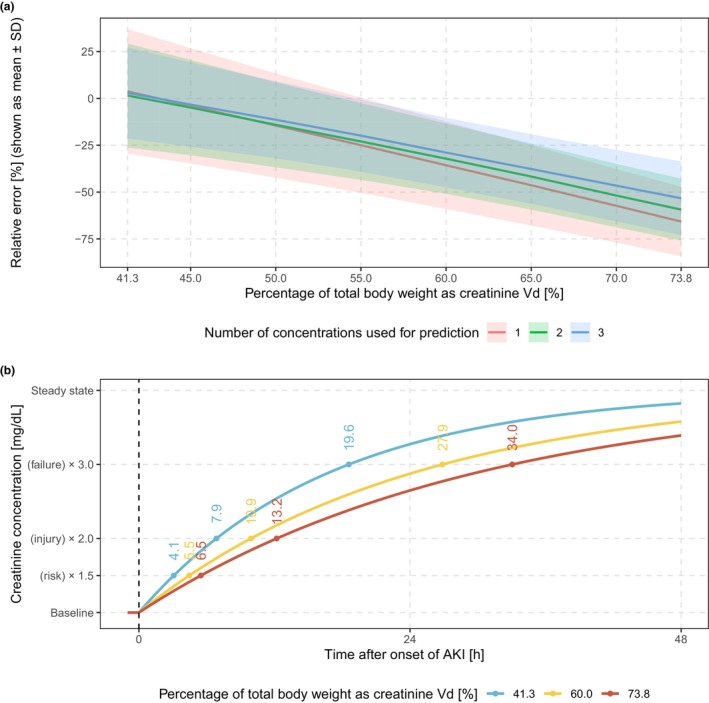
Simulations for AKI diagnosis using different values for creatinine volume of distribution (V_d_). (**a**) prediction accuracy of changed GFR after onset of acute kidney injury (AKI) comparing models with different number of concentrations. (**b**) concentration‐time curves following a 75% reduction in both GFR and nCTS and the timing to diagnose AKI risk, injury, and failure based on RIFLE criteria.

The final model was used to generate the simulation data for patients with sudden AKI. Prediction accuracy for different creatinine V_d_ settings and with 1–3 initial samples post‐AKI onset is shown in **Figure** **2** **a**. When using the reference value of 41.3%, the model captured the overall trend but was less accurate and precise with fewer concentrations. In contrast, models with V_d_ values of 60.0% and 73.8% of total body weight underestimated GFR by 35.7% and 65.7% with one concentration, by 32.2% and 59.4% with two concentrations, and by 28.9% and 53.3% with three concentrations. However, when using 2 concentrations after 24 hours and 48 hours, models showed minor differences in prediction accuracy with mean RE <1%. Concentration‐time curves following a reduction of 75% in both GFR and nCTS in one individual are illustrated in **Figure** **2** **b**. The timing of AKI diagnosis based on RIFLE criteria corresponds to the used values of creatinine V_d_ in ascending order, and the ratio of the times is approximately equal to the ratio of V_d_ used. Therefore, the timing to diagnose AKI risk (from 4.1 to 6.5 h after onset of AKI) is less dependent on the values for creatinine V_d_, but it significantly varies for definitely diagnosing kidney failure (from 19.6 to 34.0 h after onset of AKI).

### Limited sampling strategy

**Figure 3:**
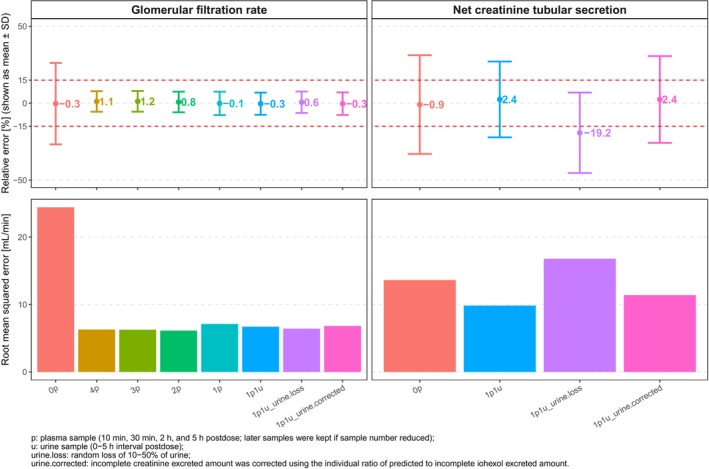
Comparison of prediction accuracy for GFR and nCTS between models using different numbers of plasma concentrations with or without urine data.

**Figure** **3** illustrates prediction accuracy for GFR and nCTS across models with varying numbers of plasma concentrations, with and without urine data. Including urine data and using more than a single plasma sample at 5 h post‐dose provided only minor improvements in GFR estimation (mean RE: −0.3 to 1.2%). However, urine data were critical for nCTS prediction accuracy, with mean RE for nCTS at −19.2% when 10–50% random urine loss occurred. Based on these findings, individual eGFR estimates derived from a single plasma sample at 5 h post‐dose were used to predict iohexol A_e_ and calculate correction factors for incomplete urine collection. Applying the correction factor to creatinine A_e_ improved nCTS prediction accuracy and reduced RMSE compared to using uncorrected “urine.loss” data.

## DISCUSSION

In this study, the dynamic creatinine model reliably described the kinetics of creatinine and essentially confirmed previous evaluations, while a lower V_d_ of 41.3% of TBW was found. Simulations to assess the impact of different V_d_ values illustrated the relevance of a proper V_d_ estimate to accurate diagnosis of AKI. nCTS, quantified by the joint creatinine/iohexol model, accounted for 31% of renal CrCL and shows promise as a tool for assessing OCT2/MATE activity *in vivo* at least in healthy individuals.

A primary objective of this study was to precisely estimate creatinine V_d_ in order to refine the creatinine PK model. Based on previous studies,^11^, ^12^ boiled beef was selected as an external source of oral creatinine to induce a perturbation in creatinine PK away from the steady‐state baseline (as with the usual single‐point method), and with frequent sampling, to allow modeling the time course of creatinine PK. Fitting the creatinine time‐course profile in plasma, along with the amounts excreted in urine, informed the estimation of the absorption rate, bioavailability, and V_d_, based on the assumption that all systemically available creatinine is excreted renally. An overall recovery of 104% for iohexol over 24‐hours post‐dose (data will be reported separately) indicated the completeness of the urine collection.^18^ However, determining the true creatinine dose was challenging due to the incomplete absorption of creatinine from beef and inconsistent differences in creatinine excretion between meat and non‐meat periods of this study over 16 and 24 hours. This discrepancy may be due to the significant interference from the large amount of endogenous creatinine. Even a small fraction of daily creatinine production (~2000 mg) could introduce substantial errors in estimating the external creatinine dose, especially when the external amount is as low as ~300 mg. Given lower residual errors in plasma data compared to urine data, simultaneous estimation of F1 and V_d_ provided the best model fit.

In the covariate assessment, TBW and estimates of the Cockcroft‐Gault equation were included as covariates based on prior knowledge.^20^ Additionally, sex was identified as a statistically significant covariate on nCTS, potentially due to a higher abundance and expression of transporters in males compared to females.^29^ However, the effect of sex on nCTS remains uncertain, as the small sample size in this study limits the robustness of this finding. Age is used as a key factor in eGFR equations but was not identified as significant in this study, likely because of the small sample size and the limited age range.^30^ The food effect on GFR and nCTS was not significant, possibly due to the limited amounts of non‐meat protein in the meals provided. A lower serum albumin level corresponding to a higher level of nCTS was observed in previous studies.^31^, ^32^ However, it was not found as a significant covariate on nCTS maybe due to only healthy participants included in the current study.

The estimated nCTS in this study accounts for approximately 31% of the total CrCL, which falls within the reported range of 10–40%.^13^ The ratios of 32.0% and 4.8% have been reported in healthy individuals in rehydrated and dehydrated states, respectively.^33^ To stimulate urine production, 240 mL of water was administered during every urine collection interval in this study, which may have kept participants in a rehydrated state and thus led to a relatively high contribution of nCTS to CrCL.

Among evaluated eGFR equations, including cystatin C did not improve predictive performance compared to creatinine‐only equations, thus failing to provide additional evidence to support the broader use of cystatin C. A possible explanation for this finding is that the study included only healthy Caucasian participants because Cystatin C has shown a greater sensitivity in patients with impaired kidney function and is less influenced by race.^34^, ^35^

The assumption that creatinine V_d_ equals to total body water (60% of TBW) has been widely used in creatinine models.^10^, ^36^, ^37^, ^38^ In contrast, the previous analysis estimated it at 73.8%,^12^ while the current study estimated it at 41.3%. These results suggested a close relationship between assumed and true values, but also highlighted potential discrepancies. Simulations (**Figure** **3**) using a final model with different V_d_ values (41.3% as a reference value) revealed significant uncertainty in predicting GFR following AKI, with substantial discrepancies observed when using “biased” V_d_ values, such as 60% or 73.8%. This highlights the critical importance of accurately selecting the V_d_, underscoring the need for caution when assuming a value for creatinine V_d_.

Previous studies have shown that 1 to 4 plasma samples within 5 hour post‐dose are sufficient to accurately estimate IoCL following a 3,235 mg iohexol dose.^24^, ^39^, ^40^ However, incomplete urine collection may result in an underestimation of CrCL, thus leading to an underestimation of nCTS.^41^ Applying a correction factor based on the ratio of predicted to observed iohexol excretion can resolve this discrepancy. This study demonstrated the feasibility of a joint model for iohexol and creatinine, using a single plasma sample at 5 hours and a urine sample from the 0–5‐hour interval after a 259 mg iohexol dose, to accurately predict both GFR and nCTS. Therefore, this approach also shows the potential for assessing renal OCT2/MATE activity based on estimated nCTS.

Apart from the limitations discussed above, renal elimination was assumed to be the sole pathway for creatinine, though minor pathways such as gut metabolism may exist.^42^ The small sample size and the narrow range of demographics in this study in healthy volunteers limited the ability to accurately assess covariate relationships and to extrapolate the results to other populations, such as the elderly or those with impaired kidney function. Early in each study period, highly variable plasma concentrations and unexplained outliers in urinary excretion were observed, likely due to the study design requiring early morning arrival at the ward, potentially sustaining elevated physiological activity. A proportional error model best fit creatinine plasma data measured via LC–MS/MS but may not apply to clinical samples measured using the Jaffe method, leading to potential bias in simulated data versus real‐world data.

In conclusion, the joint model for iohexol and creatinine, incorporating CrCL as the sum of GFR (equivalent to IoCL) and nCTS, while accounting for TBW effects and circadian variation, accurately described plasma and urine concentrations. The estimated creatinine V_d_ was 28.9 L or 41.3% of TBW. Simulations revealed significant differences in predicting GFR changes after AKI based on varying creatinine V_d_, emphasizing the importance of careful selection. Following a low‐dose iohexol administration, a single plasma and urine sample was proven sufficient to predict GFR and nCTS even for incomplete urine collection, demonstrating potential use in assessing renal OCT2/MATE activity.

## FUNDING

No funding was received for this project. Zhendong Chen and Qian Dong received scholarships from the China Scholarship Council to support their PhD studies.

## CONFLICTS OF INTEREST

The authors declared no competing interests for this work.

## AUTHOR CONTRIBUTIONS

Z.C., U.F., and M.T. wrote the manuscript; U.F., Z.C., Q.D., and M.T. designed the research; Z.C., Q.D., C.D., J.B., and U.F. performed the research; Z.C., Q.D., and M.T. analyzed the data.

## Supporting information


Data S1.
