# Supplementary material for: A Joint Pharmacometric Model of Iohexol and Creatinine Administered through a Meat Meal to Assess GFR and Renal OCT2/MATE Activity
Source: Clin Pharmacol Ther. 2025 Feb 25;118(2):510–9. doi: 10.1002/cpt.3612 (PMC12272311; doi:10.1002/cpt.3612)
Supplement: Supplementary file 1 — Data S1. [file CPT-118-510-s001.docx]

## Supplementary material

## Material and method

### Bioanalysis

The concentrations of creatinine and iohexol in plasma and urine were determined using validated liquid chromatography–mass spectrometry methods. Quantitative analysis was performed using an Agilent 1200 liquid chromatography system, which includes a binary pump and a 1260 autosampler (Agilent Technologies Deutschland GmbH, Waldbronn, Germany), coupled with an API 5000 triple quadrupole mass spectrometer equipped with an electrospray ionization source (AB Sciex Germany GmbH, Darmstadt, Germany). The data were analyzed using Analyst (version 1.6.2) software.

The lower limits of quantification for iohexol were 25 ng/mL in plasma and 125 ng/mL in urine, respectively. Inaccuracy and imprecision of were less than 15% in all cases. Details of the analysis of iohexol will be reported separately.

Creatinine was extracted from plasma and urine by protein precipitation using acetonitrile. After centrifugation, the supernatant was then separated on a ZIC-HILIC HPLC Column (5 μm, 2.1×100 mm, Merck, Germany) at a column temperature of 40°C using mobile phases of water containing 0.1% formic acid (A) and acetonitrile containing 0.1% formic acid (B). The gradient elution mode was set as follow: 0 – 0.3 min, 15%A; 0.3 – 1.0 min, 15 – 70%A; 1.0 – 2.5 min, 70%A; 2.5 – 2.51 min, 70% – 15%A; 2.51 – 4.00 min, 15%A, with a flow rate of 0.6 mL/min. The settings for IonSpray voltage and temperature were 4500 V and 400°C, respectively. The ion-pair transitions of 114.0→86.0 and 117.0→89.0 were used to monitor creatinine and d_3_-creatinine (internal standard, IS), respectively. As creatinine is an endogenous substance, methods of background subtraction and surrogate matrices were used to address the lack of blank matrices for quantification in plasma and urine, respectively.^1^ Due to relatively stable plasma concentration of creatinine (usually around 10 μg/mL), a lower limit of quantification of 2.00 μg/mL (20% of the background peak areas) was defined to achieve enough signal-to-noise ratio. ^1^ Considering much higher and broader range of urinary creatinine concentrations, deionized water was chosen as surrogate matrix. The appropriateness of using deionized water for this purpose was confirmed by assessing the creatinine to IS ratio in mixtures (*v*/*v*, 1:1) of pure creatinine and IS with six urine sample aliquots. A percentage difference within ±20% between this ratio in the mixed samples and the theoretical value is deemed acceptable as a potential matrix.^2^ Linearity was demonstrated using the least squares method based on the peak area ratios of creatinine to d_3_-creatinine, calculated with an inverse relationship (1/r). This was observed over a standard curve ranging from 2.00 to 40.0 μg/mL for plasma and from 10.0 to 1000 μg/mL for urine. Accuracy and precision were evaluated across four concentration levels: 2.00, 6.00, 15.0, and 30.0 ng/mL in plasma and 10.0, 30.0, 150, and 750 μg/mL in urine. The relative deviations for accuracy fell within the range of -14.8 to 13.0% for plasma and -13.2 to 11.0% for urine, while the coefficients of variations (CVs) for precision fell within the range of 0.9 to 6.9% and 0.7 to 7.9% for plasma and urine, respectively. Creatinine stability in plasma and urine was confirmed after four freeze-thaw cycles, at room temperature for up to 24 hours, and in a refrigerator at temperatures below −20°C. Creatinine in cooked beef was extracted using deionized water at a 5-fold weight ratio. The extraction was processed and measured in the same way applied to urine samples.

### Non-compartmental analysis

Creatinine and iohexol amounts excreted in urine were calculated using respective concentrations and the documented urine volumes collected at each interval. Individual iohexol clearance (IoCL) and creatinine clearance (CrCL) were then determined by dividing the excretion amounts by the area under the plasma concentration-time curve (AUC) for each substance. Mean IoCL and CrCL were calculated for each participant over the respective periods. A one-way analysis of variance (ANOVA) was used to assess significant differences in iohexol and CrCL across different periods. Urine flow rates were calculated by dividing the urine volume by duration of the collection interval. Regression analysis examined the relationships between IoCL, CrCL, urine flow, and urine pH at each interval.

## Results

### Non-compartmental analysis

Individual plasma concentration-time curves for creatinine in three periods are shown in Fig. S6. Relatively constant creatinine plasma concentrations were obtained during non-meat periods. In the pilot study participants 1 and 2 showed minimal changes after consuming beef cooked at 70°C, suggesting higher cooking temperature is crucial for producing creatinine in beef as creatinine is a degradation product of creatine. Therefore, a temperature of 90°C was used in the main study, where clear increased creatinine concentrations were observed following beef ingestion in participants 003 – 014. Fig. S7 displays the individual IoCL and CrCL at each urine collection interval, both exhibiting similar diurnal patterns, with higher values during the day and lower at night.

All four variables – IoCL, CrCL, urine flow, and urine pH – showed correlations (Fig. S8). An expected correlation (r = 0.799) between IoCL and CrCL was observed with a positive relationship. Additionally, urine flow and urine pH exhibited a moderate positive correlation (r = 0.501), while urine flow and CrCL showed a weak positive correlation (r = 0.274). Regression plots suggest that urine flow increased with higher CrCL and urine pH, particularly for CrCL <125 mL/min and urine pH <6.5.

Statistical results, including baseline creatinine concentration, daily creatinine excretion, IoCL, and CrCL over three periods, are shown in Table S3. Baseline creatinine concentrations were consistent with the values from screening values. Daily creatinine excretion during non-meat periods showed no significant changes. No significant differences were observed in either IoCL (*p* = 0.31) or CrCL (*p* = 0.67) between periods, based on one-way ANOVA analysis. These results indicate that the creatinine production and kidney function of the participants did not change significantly during the study.

## References

1. Thakare R, Chhonker YS, Gautam N, Alamoudi JA, Alnouti Y. Quantitative analysis of endogenous compounds. J Pharm Biomed Anal. 2016 Sep 5;128:426-437.
2. Ou M, Song Y, Li S, Liu G, Jia J, Zhang M, Zhang H, Yu C. LC-MS/MS Method for Serum Creatinine: Comparison with Enzymatic Method and Jaffe Method. PLoS One. 2015 Jul 24;10(7):e0133912.

## Supplementary tables

Table S1 Comparison of model estimates between separate and joint model for iohexol and creatinine.

| **Parameters** | Estimate (RSE%) |  |
| --- | --- | --- |
|  | Separate models | Joint model |
| **Fixed effect** |  |  |
| **Iohexol** |  |  |
| GFR (mL/min) | 95.2 (4.2) | 95.2 (3.8) |
| V_c_ (L) | 9.54 (10.9) | 9.55 (4.1) |
| Q_p1_ (L/h) | 0.179 (18.4) | 0.179 (10.6) |
| V_p1_ (L) | 1.44 (11.1) | 1.44 (6.3) |
| Q_p2_ (L/h) | 4.39 (49.2) | 4.38 (12.3) |
| V_p2_ (L) | 4.43 (25.7) | 4.43 (6.9) |
| **Creatinine** |  |  |
| K_a_ (1/h) | 1.58 (13.8) | 1.58 (15.1) |
| CrCL or nCTS (mL/min) | 132 (6.2) | 35.7 (15.5) |
| V_d_ (L) | 26.0 (9.9) | 26.0 (14) |
| F1 (%) | 0.466 (8.9) | 0.466 (6.9) |
| Lag time (h) | 0.289 (3.9) | 0.289 (3.4) |
| CGR (mg/h) | 66.8 (20.1) | 66.8 (9.8) |
| **Random effect (IIV)** |  |  |
| **Iohexol** |  |  |
| GFR | 0.0204 (44.8) | 0.0209 (47.7) |
| V_c_ | 0.0333 (76.9) | 0.0339 (105.6) |
| V_p1_ | 0.00683 (59.2) | 0.00697 (43.8) |
| V_p2_ | 0.0241 (40.7) | 0.0249 (34.8) |
| **Creatinine** |  |  |
| CrCL or nCTS | 0.0416 (33.2) | 0.185 (34.4) |
| V_d_ | 0.0271 (53.5) | 0.0260 (73.5) |
| F1 | 0.0167 (68.3) | 0.0165 (145) |
| CGR | 0.0914 (59.1) | 0.0885 (31.0) |
| **Random effect (RV)** |  |  |
| **Iohexol** |  |  |
| Plasma concentration | 0.0177 (18.5) | 0.0177 (20.2) |
| Excreted amount in urine | 0.0611 (29.6) | 0.0611 (36.8) |
| **Creatinine** |  |  |
| Plasma concentration | 0.00286 (8.9) | 0.00286 (9.6) |
| Excreted amount in urine | 0.0424 (43.9) | 0.0426 (45.5) |

GFR, iohexol clearance was assumed as GFR; CrCL, creatinine clearance; nCTS, net tubular secretion part of CrCL; V_c_, iohexol central compartment volume; Q_p1_, inter-compartment clearance between central and first peripheral compartment; V_p1_ first peripheral compartment volume; Q_p2_, inter-compartment clearance between central and second peripheral compartment; K_a_, apparent absorption rate; V_d_, creatinine volume of distribution; CGR, creatinine generation rate; F1, bioavailability; RSE, relative standard error; CV, coefficient variance; IIV, inter-individual variability; RV, residual variability.

Table S2 Additional attempts for covariate analysis on clearance (CL) and volume of distribution (V).

| Model no. | Covariate | Reference model | OFV change |
| --- | --- | --- | --- |
| 1 | TBW + Fixed values*^a^* | - | - |
| 2 | FFM***^b^*** + Fixed values | 1 | 7.47 |
| 3 | FFM***^b^*** + Fixed values + FAT + estimating FAT effect | 1 | 0.82 |
| 4 | TBW + estimating scaling factors for CL | 1 | -0.853 |
| 5 | TBW + estimating scaling factors for V | 1 | -0.836 |

OFV, objective function value; fixed values, fixed scaling factors (0.75 for CL and 1.0 for V); TBW, total body weight; FFM, free fat mass; FAT, difference between TBW and FFM.

*^a^*González-Sales M, Holford N, Bonnefois G, Desrochers J. Wide size dispersion and use of body composition and maturation improves the reliability of allometric exponent estimates. J Pharmacokinet Pharmacodyn. 2022 Apr;49(2):151-165.

*^b^*Holford NHG, Anderson BJ. Allometric size: The scientific theory and extension to normal fat mass. Eur J Pharm Sci. 2017 Nov 15;109S:S59-S64.

Table S3 Statistic results from non-compartmental analysis. Data are shown as mean (SD).

| **Period***^a^* | **N** | **Creatinine concentration at baseline (mg/dL)***^b^* | **Creatinine excreted amount in 24 h (mg)** | **Iohexol clearance (mL/min)*^c^*** |  | **Creatinine clearance (mL/min)*^c^*** |  |
| --- | --- | --- | --- | --- | --- | --- | --- |
| Reference | 12 | 0.902 (0.192) | 1602 (601) | 87.2 (23.0) |  | 127 (33) |  |
| Test | 14 | 0.876 (0.139) | 1609 (526) | 98.2 (18.5) |  | 134 (32) |  |
| Meat | 14 | 0.892 (0.159) | 1941 (487) | 98.7 (21.2) |  | 139 (33) |  |
| All | 14 | 0.890 (0.160) | 1606 (550)*^d^* | 95.1 (21.0) |  | 133 (32) |  |

*^a^*Participants received iohexol doses of 3259 mg and 259 mg in reference and test periods, respectively, and received a 3259 mg dose of iohexol along with meat intake during the meat period.

*^b^*Creatinine plasma concentration was measured by LC-MS/MS

*^c^*Clearance was calculated by dividing respective AUC by excreted amount in urine.

*^d^*Result was calculated based on data only from non-meat periods.

## Supplementary figures


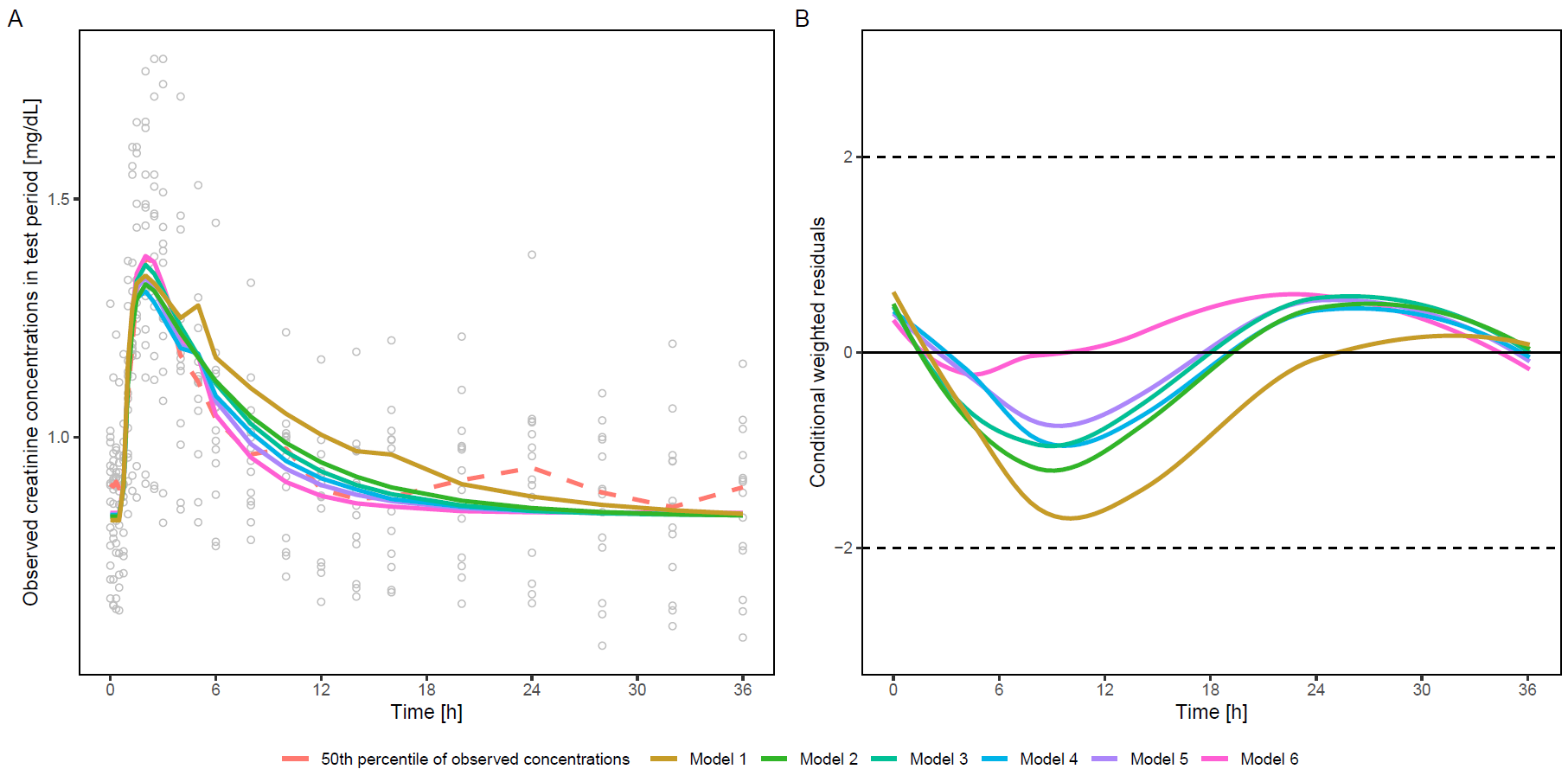


Fig. S1 Comparison of population predictions (A) and conditional weighted residuals (B) between models with different settings (Table 1) for dose input, bioavailability, and volume of distribution for creatinine.

Fig. S2 Schematic diagram of the joint model. Following bolus administration to the central compartment (volume V_c_), iohexol is distributed into two peripheral compartments (volumes V_p1_ and V_p2_) in parallel, with inter-compartment clearances (Q_p1_ and Q_p2_). It is eliminated from the central compartment via glomerular filtration rate (GFR). Creatinine, derived from the meat meal, enters the central compartment (volume V_d_) via first-order absorption (Ka) with bioavailability (F1). Simultaneously, creatinine is continuously produced at a constant rate (CGR) within the central compartment and is eliminated through both GFR and the net creatinine tubular secretion (nCTS), accounting for reabsorption.


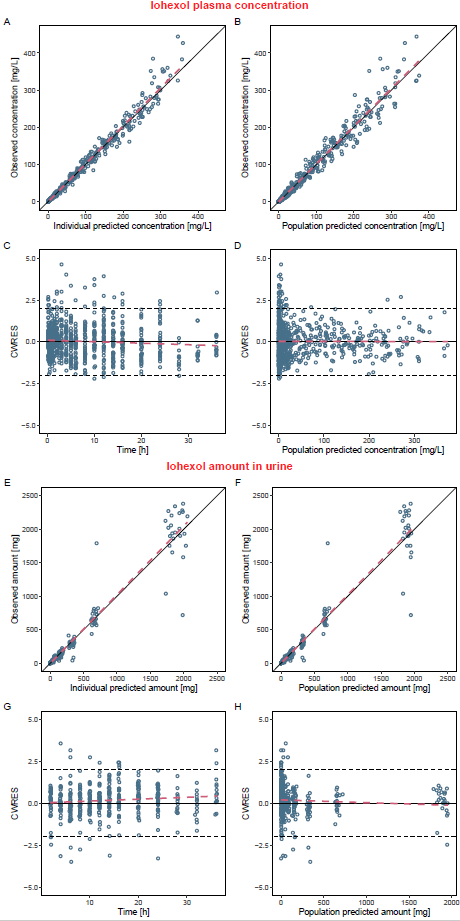


Fig. S3 Combined goodness of fit plots of the final model for iohexol. CWRES, conditional weighted residuals; Time, time after iohexol administration. Red lines show the linear model fit.


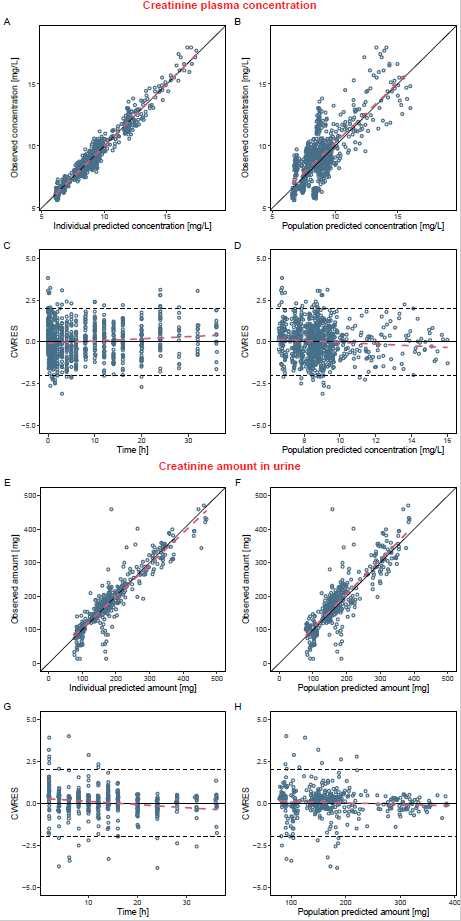


Fig. S4 Combined goodness of fit plots of the final model for creatinine. CWRES, conditional weighted residuals; Time, time after iohexol administration. Red lines show the linear model fit.


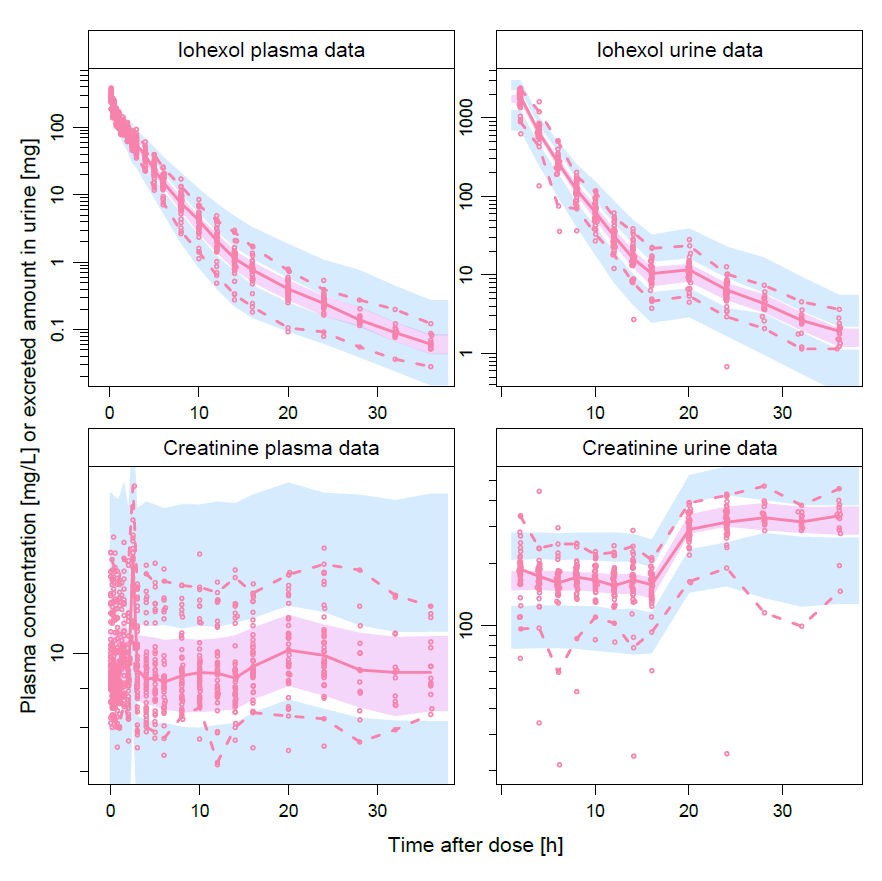


Fig. S5 Predictive correlated visual predictive check (*n* = 1000) stratified by substance for the final model for plasma and urine. Dots represent observed concentrations. Solid lines represent the median values, while dashed lines show the 5^th^ and 95^th^ percentiles of observed concentrations. Shaded areas are the model-predicted 95% confidence intervals for the 5^th^ (blue), 50^th^ (pink), and 95^th^ (blue) percentiles from 1000 simulated data sets. The time of urine data is the end each collection interval.


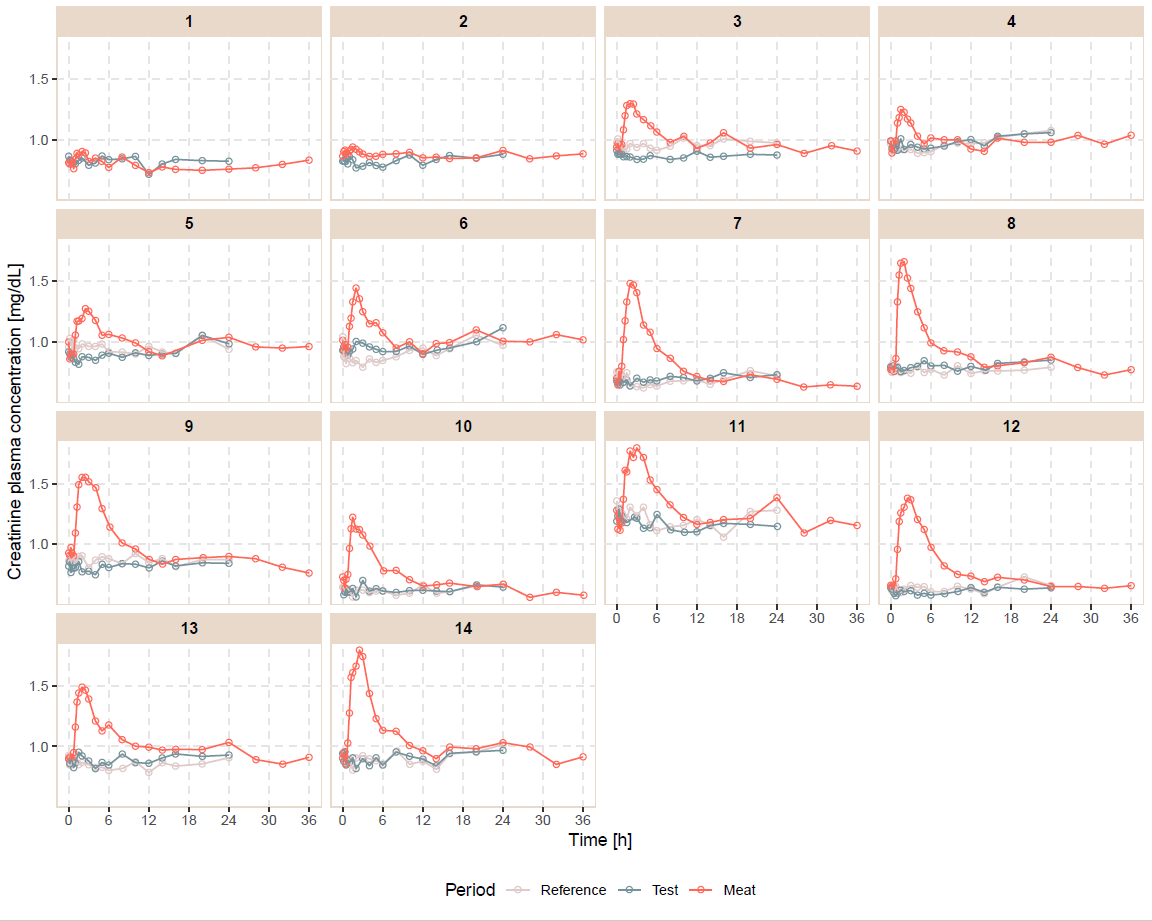


Fig. S6 Individual plasma concentration-time curves of creatinine in reference, test, and meat periods (*n* = 14).


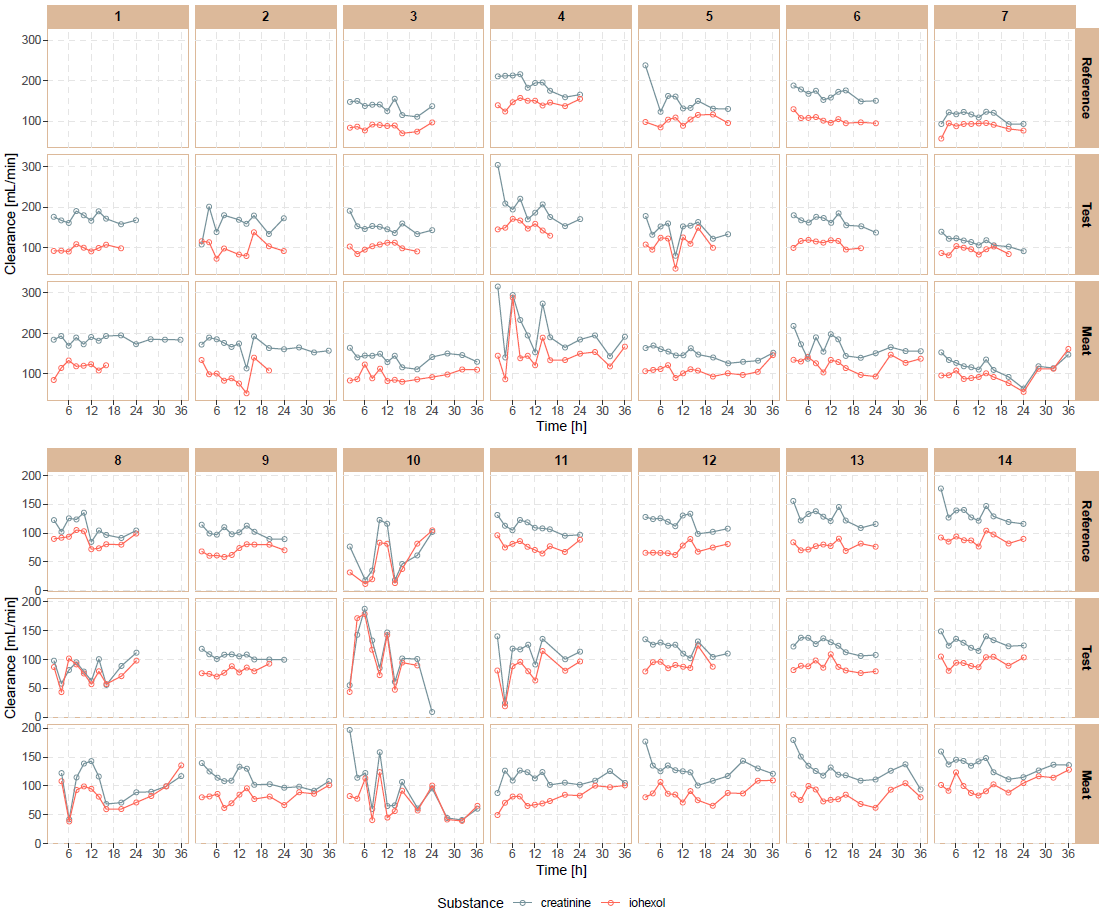


Fig. S7 Individual creatinine and iohexol clearances calculated at each time interval by non-compartmental methods in reference, test and meat periods (*n* = 14).


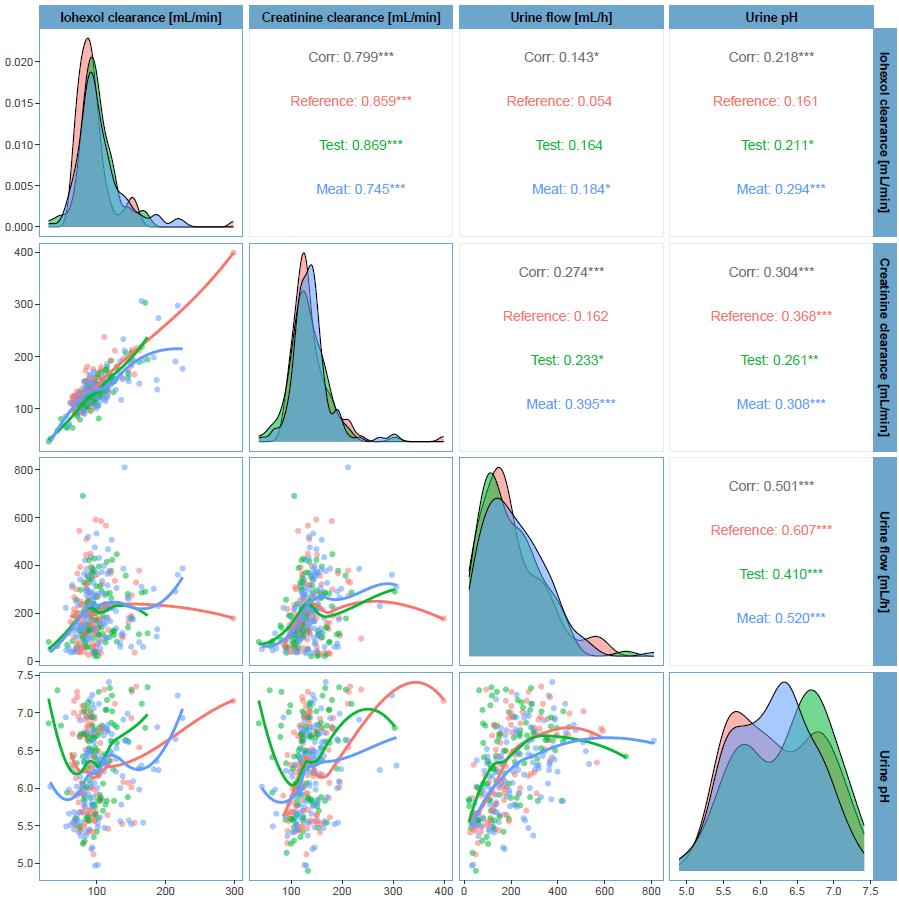


Fig. S8 Correlations between iohexol clearance, creatinine clearance, urine flow, and pH value at each urine collection interval stratified by period. *, **, and *** denote statistical significance at p-values < 0.05, < 0.01, and < 0.001, respectively.

## NONMEM code

$PROBLEM

$INPUT ID TIME DV AMT MDV CMT EVID AGE TBW SEX

$DATA LOWIO_NM_2_FFM.csv IGNORE=@

$SUBROUTINE ADVAN13 TOL=6

$MODEL NCOMP=7 COMP=(CENTRAL_IOX) COMP=(CENTRAL_CRE)

COMP=(URINE_CRE) COMP=(URINE_IOX) COMP=(DEPOT_BEEF)

COMP=(PERI1_IOX) COMP=(PERI2_IOX)

$PK

; Covariate relationship

SEXonTS=(THETA(15)**(1-SEX))

CG=((140-AGE)*TBW/72)*(0.85**(1-SEX))*60/100; *60/100 is to convert unit from ml/min*mg/dL to mg/h

TBWonCL=((TBW/70)**0.75)

TBWonV=(TBW/70)**1

; Circadian rhythm;

IF(TIME.LT.24)THEN

DAY = 1

ELSE

DAY = 2

ENDIF

PI=3.141592653

IF(TIME.GE.14.AND.TIME.LE.24)THEN

TVRATIO = THETA(14) ;Night

RATIO = TVRATIO

CIR = -SIN((TIME-14)/10*PI)*RATIO+1

ELSE

TVRATIO = THETA(13) ;Daytime

RATIO = TVRATIO

CIR = SIN((TIME-(DAY-1)*24)/14*PI)*RATIO+1

ENDIF

; Creatinine parameters

TVKA5 = THETA(1)

KA5 = TVKA5*EXP(ETA(1))

TVCL_SEC = THETA(2)

CL_SEC = TVCL_SEC*EXP(ETA(2))*CIR*TBWonCL*SEXonTS

TVV_CRE = THETA(3)

V_CRE = TVV_CRE*EXP(ETA(4))*TBWonV

TVF5 = THETA(4)

F5 = TVF5*EXP(ETA(8))

TVCGR = THETA(5)*CG

CGR = TVCGR*EXP(ETA(6))

TVALAG5= THETA(6)

ALAG5 = TVALAG5*EXP(ETA(7))

;Iohexol parameters

TVCL_IOX = THETA(7)

CL_IOX = TVCL_IOX*EXP(ETA(3))*CIR*TBWonCL

TVV_IOX = THETA(8)

V_IOX = TVV_IOX*EXP(ETA(5))*TBWonV

TVQ1_IOX = THETA(9)

Q1_IOX = TVQ1_IOX*EXP(ETA(9))*TBWonCL

TVV2_IOX = THETA(10)

V2_IOX = TVV2_IOX*EXP(ETA(10))*TBWonV

TVQ2_IOX = THETA(11)

Q2_IOX = TVQ2_IOX*EXP(ETA(11))*TBWonCL

TVV3_IOX = THETA(12)

V3_IOX = TVV3_IOX*EXP(ETA(12))*TBWonV

K10 = CL_IOX/V_IOX

K16 = Q1_IOX/V_IOX

K61 = Q1_IOX/V2_IOX

K17 = Q2_IOX/V_IOX

K7 = Q2_IOX/V3_IOX

CL_CRE = CL_IOX + CL_SEC

K_CRE = CL_CRE/V_CRE

C0 = CGR/CL_CRE

A_0(2) = C0*V_CRE

$DES

; Creatinine

DADT(5)= -KA5*A(5) ;depot for beef ingestion

DADT(2)= KA5*A(5)-K_CRE*A(2)+CGR ;Plasma cre amount

DADT(3)= K_CRE*A(2) ;urine cre

; Iohexol

DADT(1)= -K10*A(1)-K16*A(1)+K61*A(6)-K17*A(1)+K71*A(7);Plasma iox amount

DADT(4) = K10*A(1) ;urine iox

DADT(6) = K16*A(1)-K61*A(6) ;peripheral-1 iox

DADT(7) = K17*A(1)-K71*A(7) ;peripheral-2 iox

$ERROR

IF(CMT.EQ.2) THEN ; Creatinine plasma concentrations

IPRED = A(2)/V_CRE

IRES = DV-IPRED

IWRES=IRES/(SQRT(IPRED**2*SIGMA(1,1)+SIGMA(2,2)))

Y = IPRED*(1+EPS(1))+EPS(2) ;

ENDIF

IF(CMT.EQ.3)THEN ; Creatinine urine amount

IPRED = A(3)

IRES = DV-IPRED

IWRES=IRES/(SQRT(IPRED**2*SIGMA(3,3)+SIGMA(4,4)))

Y = IPRED*(1+EPS(3))+EPS(4) ;

ENDIF

IF(CMT.EQ.1)THEN ; Iohexol plasma concentrations

IPRED = A(1)/V_IOX

IRES = DV-IPRED

IWRES=IRES/(SQRT(IPRED**2*SIGMA(5,5)+SIGMA(6,6)))

Y = IPRED*(1+EPS(5))+EPS(6) ;

ENDIF

IF(CMT.EQ.4)THEN ; Iohexol urine amount

IPRED = A(4)

IRES = DV-IPRED

IWRES=IRES/(SQRT(IPRED**2*SIGMA(7,7)+SIGMA(8,8)))

Y = IPRED*(1+EPS(7))+EPS(8) ;

ENDIF

$THETA

(0,1.70996); 1. KA_CRE

(0,2.38177); 2.CL_SEC

(0,28.943); 3.V_CRE

(0,0.523045); 4.F1_beef

1 FIX; 5.CGR

(0,0.291197); 6.LAG_CRE

(0,5.22317); 7.CL_IOX

(0,8.69091); 8.V1_IOX

(0,0.130821); 9.Q_IOX

(0,1.15193); 10.V2_IOX

(0,4.00936); 11.Q2_IOX

(0,4.21713); 12.V3_IOX

(0,0.0370398); 13.CIR_DAY

(0,0.0842088); 14.CIR_NIGHT

0.628122; 15. SEXonTS

$OMEGA

0 FIX; 1. KA_CRE

0.0519899; 2. CL_SEC

0.0140315; 3. CL_IOX

0.0226214; 4. V_CRE

0.0211092; 5. V1_IOX

0.0160459; 6. CGR

0 FIX; 7. LAG_CRE

0.0107643; 8. F1_beef

0 FIX; 9. Q_IOX

0.012076; 10.V2_IOX_

0 FIX; 11.Q2_IOX_

0.0113318; 12.V3_IOX_

$SIGMA

0.00255091; prop_CRE_plasma.

0 FIX

0.041298; prop_CRE_urine.

0 FIX

0.0171037; prop_IOX_plasma.

0 FIX

0.061672; prop_IOX_urine.

0 FIX

$ESTIMATION METHOD=1 INTER PRINT=3 MAXEVAL=9999 NSIG=2 SIGL=6 NOABORT

$COVARIANCE

$TABLE ID TIME CL_IOX CL_CRE SEC_RATIO CMT AMT MDV EVID IPRED PRED IRES CWRES IWRES ERES NOPRINT ONEHEADER FILE=sdtab92

$TABLE ID CL_SEC CGR V_CRE CL_IOX V_IOX V2_IOX V3_IOX Q1_IOX Q2_IOX PERI SEC_RATIO PERI CG ETAS(1:LAST) NOPRINT ONEHEADER FILE=patab92

$TABLE ID AGE TBW HT SEX NOPRINT ONEHEADER FILE=cotab92
